# Transforming the Process of Recognition of Medical Specialist in the Ministry of Health: A Shift from Specialist Gazettement to Supervised Work Experience

**DOI:** 10.21315/mjms-08-2025-s01

**Published:** 2025-08-30

**Authors:** Hirman Ismail, Mohd Azman Yacob, Mohamed Hirman Abdullah, Ahmad Badruridzwanullah Zun, Sabrizan Osman, Siti Baizura Amran, Nur Ainina Idris, Farah Ruwaida Fakhrul Ruzi, Siti Norsyazwanis Jalaluddin, Ahmad Yamany Othman, Nora Eliza Abdul Halim, Nor Akmal Hakim Kamarulzaman

**Affiliations:** Medical Development Division, Ministry of Health, Putrajaya, Malaysia

**Keywords:** healthcare system, human resource for health, Medical Act 1971, medical education, medical specialty, Ministry of Health, specialist training

## Abstract

The supervised practice of newly credentialed medical practitioners within the public sector following completion of specialist training, referred to previously in Malaysia as specialist gazettement or post-qualification working experience (PQWE), is a unique regulatory mechanism aimed at safeguarding the quality and safety of specialist care. This probationary phase enables senior consultants or specialists to assess the competence and readiness of the practitioners for independent clinical practice. In response to evolving demands on healthcare delivery and workforce governance, the Ministry of Health has introduced a series of strategic reforms to strengthen its framework, especially within the public healthcare setting. These include legislative amendments to formalise and regularise the implementation of the probationary phase through supervised work experience or SWE (legal transformation), fiscal measures to support financing incentives (financial transformation), and digital innovations that enhance experience and administrative efficiency (digital transformation). This paper describes these transformative interventions, positioning the Ministry of Health’s approach within broader policy discourses on clinical governance and health system sustainability.

## Introduction

The Health White Paper (HWP), passed by the Malaysian Parliament on 15 June 2023, sets a comprehensive 15-year roadmap to reform Malaysia’s healthcare system. The HWP is not just aspirational, but it is a call for systemic reform, stakeholders’ involvement, and system resilience. The HWP outlines four major pillars, and one of the pillars focuses on strengthening governance and health system’s foundations (1). This includes restructuring the role of the Ministry of Health, enhancing legislative frameworks to promote accountability and transparency, and investing in health workforce to sustain expertise and retain talent. It also emphasises the importance of data analytics and innovation, thus laying the foundation for a more responsive and future-ready healthcare system.

The amendments to the Medical Act 1971 [Act 50] in 2024 to some degree would support the HWP aspirations particularly from the perspective of strengthening governance and maintaining expertise in the system. The Medical (Amendment) Act 2024 [Act A1729] and the Medical Regulations 2025 have set a new benchmark to strengthen the governance of matters related to medical professions such as recognition of specialist training programmes and registration of medical specialist. One of the key enhancements in the process of specialist registration especially for practitioners trained locally is the formalisation of post-training probationary phase, previously known as specialists gazettement under the public sector or post-qualification working experience (PQWE). The probationary phase is now known as supervised work experience or SWE.

**Figure 1:**
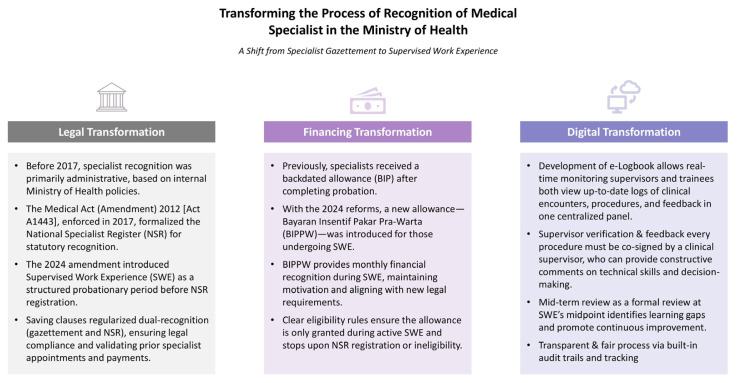
Summary of transformative initiatives in the process of specialist recognition in the Ministry of Health

Compared to models in other countries, Malaysia’s approach is uniquely designed and executed. Countries such as the United Kingdom, Australia, and the United States typically provide specialist recognition immediately upon certification of completion of training, without a legally mandated post-training probation. Ministry of Health’s SWE framework, empowered by law, financial incentives and operational support through digital platform (Figure 1), signify a distinct model in health workforce governance, aiming at ensuring quality and safeguarding patients’ safety.

## Legal Transformation

The Medical Act 1971 [Act 50], enacted on 1 October 1971, initially lacked specific provisions for the registration of medical specialists under the Malaysian Medical Council (MMC) (2). This legislative gap persisted until the Act was amended through the Medical Act (Amendment) 2012 [Act A1443]. Although Act A1443 was passed in 2012, it only came into force five years later, on 1 July 2017. Upon its implementation, the registration of specialists under a specific register or better known as the National Specialist Register (NSR) was formalised.

The NSR was firstly developed through a strategic collaboration between the Ministry of Health and the Academy of Medicine of Malaysia, with the shared objective of consolidating recognition mechanisms and strengthening transparency and public trust in specialist credentials. While the Academy had maintained its own Specialist Register since 2000 (3), the NSR was envisioned as a more comprehensive platform to serve the broader healthcare system. The enactment of the Act A1443 enabled this vision by conferring statutory authority upon the NSR through the MMC, empowered it from an administrative and voluntary initiative to a formal regulatory requirement. It has empowered NSR to discharge its functions with a higher level of credibility and accountability.

Before this statutory inclusion, specialist recognition within the public sector relied heavily on administrative mechanisms governed by internal policy instruments. One of the key instruments was the General Orders in Chapter F: on medical affairs or “Perubatan,” which governs entitlements, responsibilities, and formal appointments for specialists and other matters related to medical affairs in the civil service. This Chapter was officially issued under the authority of the Yang di-Pertuan Agong (the King of Malaysia), pursuant to Clause (2), Article 132 of the Federal Constitution, and came into force on 1 March 1974, effectively repealing the 1957 version (4).

Under General Order 27 of Chapter F, the Director General of Health holds discretionary authority to appoint medical officers as specialists. Such appointments are made based on the advice of three appointed specialists or also known as Special Medical Committee or “Jawatankuasa Khas Perubatan (JKP)” established by the Government (2, 4). The General Orders stipulated that a medical officer could only be appointed as a specialist if the role they held required specialist-level functions or duties. Additionally, the officer must possess either recognised academic or professional qualifications and relevant experience deemed satisfactory by the Director General or have demonstrated consistent and satisfactory performance of specialist-level functions over a significant period (4). Names of all specialists in the public sector appointed by the Director General were published through Government Gazette and therefore such process of appointment was also known as specialist gazettement.

Following the enforcement of Act A1443 in 2017, it remained a practice for medical practitioners who had completed specialist training to first undergo a probationary period and be appointed as specialists by the Director General of Health under General Order 27, prior to their formal registration in the NSR under the MMC. This dual pathway of specialist recognition, via administrative gazettement under General Orders and statutory registration under the NSR, has created an institutional dichotomy.

The public healthcare sector historically relied on the authority granted under General Order 27 to appoint medical officers as specialists, allowing them to assume specialist-level duties and receive corresponding remuneration such as specialist allowances or also known as the Bayaran Insentif Pakar. Meanwhile, the NSR, introduced as a formal register following the Act A1443, became the legally recognised means for specialist registration.

This resulted in a sequential and systemic dual-recognition process: first, a public sector medical officer would complete specialty training and be appointed a specialist by the Director General of Health, effectively recognised within the civil service (or gazettement) and second, only subsequent to the gazettement would they apply for registration with the NSR, which is the statutory prerequisite to practise as a specialist under Act 50.

The discussion about these two recognitions became evident following legal clarification during the 2024 amendment to the Medical Act. Specialist recognition before NSR registration was partially aligned with the provisions of the amended law in the year 2012. Section 14A of the Act 50 stated that “No person whose name has not been entered into the register shall practise as a specialist in that specialty” (5).

In the latest amendment to the Medical Act in 2024 [Act A1729] (6), the concept of SWE was introduced under Subparagraph 14B(1)(d) (i), to streamline the prior specialist gazettement probationary phase. The structured post-training probationary phase in SWE is somewhat similar to the process of gazettement probationary period, during which the practitioner continues to perform specialist-level duties under supervision. Registration with the NSR may only occur after satisfactory completion of SWE, aligning with Section 14B and 14C of Act A1729.

The Medical (Amendment) Regulations 2025 [P.U. (A) 198], which came into force on 1 July 2025, has stipulated that any medical practitioner serving in the public sector must first be validated by the Director General of Health as having successfully completed the SWE before being eligible for evaluation and registration in the NSR by the Evaluation Committee for Specialist Medical Qualifications under the MMC. The transformation of legal provisions and administrative processes has regularised the recognition and registration of medical specialists within the public service, ensuring better alignment with statutory requirements and legal standards.

To regularise the processes and recognition of specialists, particularly within the public sector prior to the 2024 amendment of the Medical Act 1971, saving clauses were introduced under subsection 11(6) of the Act A1729. This provision stipulated that any individual who was appointed as a specialist by the Director General of Health, or in other words, gazetted under General Order 27, shall be deemed to be registered as a specialist under the Act. The saving clause has legally regularised the so-called dual recognition process of specialist recognition as practised before the enforcement of the amended Medical Act in the year 2024. Furthermore, any remuneration, allowances, or payments disbursed to such individuals in their capacity as specialists were validated and declared to have been lawfully paid as stipulated in Section 12 of the Act A1729 (6).

## Financial Transformation

Financial transformation in the context of specialist recognition refers to the Ministry of Health’s revisions to policies and procedures governing financial incentives for medical practitioners undergoing SWE. These changes were implemented in conjunction with the enforcement of Act A1729. Prior to Act A1729, the Specialist Incentive Allowance, known as Bayaran Insentif Pakar (BIP), was given to practitioners who had completed their specialist gazettement process.

The incentive was issued as a cumulative payment, retrospectively covering the entire period from the initial date of gazettement until official certification of completion. This approach, commonly referred to as “backdated allowance,” meant that practitioners received their financial incentives only after successful certification of the probationary phase, with payments reflecting the full duration of their specialist gazettement process. The cumulative retrospective allowance arrangements were implemented since 2014 following a meeting between the Public Service Department and the Ministry of Health.

Practitioners undergoing SWE deserve continued financial recognition due to the substantive nature of clinical tasks they perform throughout this transitional or probationary phase. Although not formally registered as specialist, they operate or function with specialist-level accountability such as performing surgeries, leading clinical management in wards or critical care areas, delivering diagnostics, and responding to emergencies. Their clinical outputs are often indistinguishable from those of certified or registered specialists, reflecting certain degree of autonomy and decision-making in clinical setting. The only characteristic that differentiates them is the supervisory requirement by the more senior specialist during the SWE. These practitioners are often redeployed to high-demand, remote or unpopular facilities during SWE, amplifying workload and social strain.

SWE also demands rigorous and detailed documentation, including mandatory clinical procedural logbooks and evaluation through a supervisor narrative report, covering eight competency domains with threshold scores for progression. The administrative and professional intensity of SWE underscores the fairness of financial reward or incentives. Due to this demanding transitional probational phase, the Ministry of Health strongly believed that withholding financial incentives merely due to NSR registration status was somewhat counterproductive. Following extensive consultations with the Public Service Department, the Ministry introduced a special allowance for practitioners undergoing SWE or known as the Bayaran Insentif Pakar Pra-Warta (BIPPW) to ensure continued recognition (7), alignment with regulatory reforms, and sustained motivation in the specialist recognition pathway.

The BIPPW framework is a strategic solution to potential discontinuity of cumulative retrospective BIP payments following the enforcement of the Act A1729. Effective 1 July 2025, BIPPW applies to officers in the Ministry of Health, Malaysian Armed Forces, and Public Universities governed by the public service remuneration system. The rate was matched to the existing BIP allowance based on individual salary grade. By institutionalising this financial incentive during SWE, the Ministry of Health safeguarded morale and retention amidst increasing complexity in specialist career and professional progression. The policy also addressed legal reconciliation by supporting the regularised process of specialist recognition through the SWE and NSR registration especially within the public sector. Such systematic and consequential legal process which is quite unique to the Malaysian healthcare system requires clear policy mechanisms like BIPPW to uphold consistency and regularity across recognition, appointment and remuneration package.

To ensure financial accountability, clear eligibility and withdrawal criteria govern the implementation of the allowance. BIPPW is granted from the initiation of SWE and would be terminated upon NSR registration or due to other reasons such as unsuccessful SWE certification, delay in documentation submission, extended leave, or re-assignment to non-core duties, as outlined in the relevant circular of the Public Service Department. The Ministry opted for monthly disbursement of the BIPPW allowance, rather than cumulative retrospective payments as previously practised, further regularised the administrative financial procedure. SWE failure rate was relatively very low, on average at only less than 1% in the past 10 years, further validating the BIPPW policy’s cost-efficiency and targeted design.

**Figure 2:**
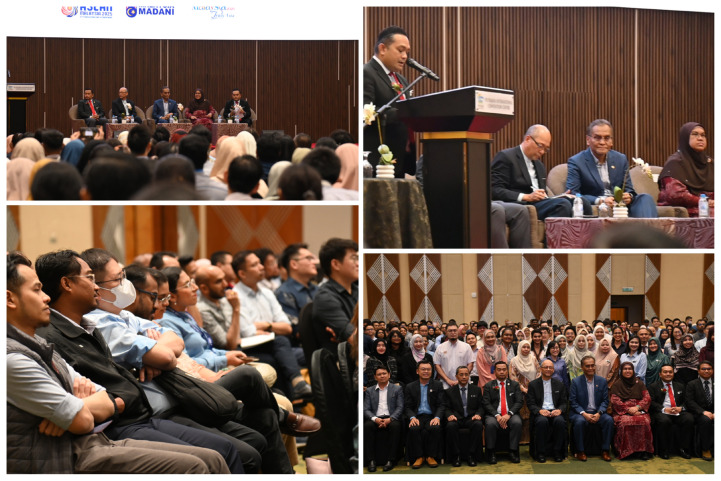
Briefing session on 14 July 2025 with the pioneering cohort of 402 medical practitioners undergoing SWE following the completion of their specialist training, where the approval of allowance was announced by the Minister of Health

Although there’s always a possibility of a higher failure and extension rates of SWE due to a more robust monitoring mechanism, which will be explained in the next section, and due to higher competency standards, the number is still expected to be relatively low compared to the overall annual number of practitioners completing specialist training through both master’s and parallel pathway programmes. The approval of the BIPPW allowance was officially announced by the Minister of Health on 14 July 2025 (8–10) during a briefing session with the pioneering cohort of 402 medical practitioners undergoing SWE following the completion of their specialist training (Figure 2).

## Digital Transformation

Digital transformation in the process of specialist recognition refers to the initiatives by the Medical Development Division to digitalise the process of probationary phase or now known as SWE through the Medical Programme Information System. In the previous editorial, the role of MPIS was described as a mean to improve efficiency in the various processes relating to hospital management under the Medical Programme of the Ministry of Health. MPIS is a cloud-native web application initially designed during the COVID-19 pandemic to monitor hospital capacity and preparedness. It has since evolved into a comprehensive platform comprising multiple modules, including the Specialist Database Module, which plays a central role in mapping the distribution of medical specialists across the Ministry of Health hospitals. At the time of writing, MPIS has been expanded to include seven modular components. These components include, i) Asset Management, which facilitates medical equipment applications, and multi-tiered verification and approval processes; ii) Clinical Surveillance, which manages mortality registration, verification, announcements, and mortuary functions; iii) Clinical Process Management, encompassing admission-discharge-transfer, outpatient and ward management, clinical orders; iv) Hospital Documentation, which includes medical record handling, report generation, and medical board documentation; v) Healthcare Worker (HCW) Management and Development, covering data and processes related to medical assistants, nurses, house officers, and specialists; vi) Helpdesk, which supports service ticketing and quick board functions for operational troubleshooting; and vii) Programme Management, which oversees system and user administration, hospital capacity tracking, billing, transplant services, dashboard analytics, and audit trails. Under the Healthcare Worker (HCW) Management and Development component, a special section on the certifying process and logbook on the probationary phase or SWE was developed.

**Figure 3:**
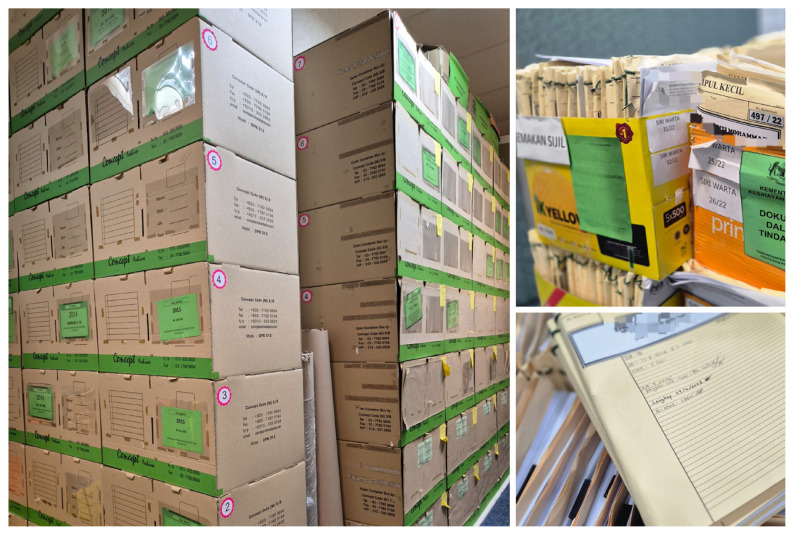
Exhibits to demonstrate the burden of paper handling in the process of specialist gazettement

The specialist gazettement process posed such a significant administrative challenge. The process was entirely paper-based and relied on hospitals forwarding over a thousand sets of hardcopy documents each year. Every document set, ranging from appointment letters, logbooks, administrative profiles to narrative reports, were processed and verified physically through hospital human resource units, the secretariat, panels of Special Medical Committee (JKP), and finally to the Director General. This created a heavy administrative burden, as staff repeatedly handled, recorded, and re-entered data into separate spreadsheets and files, consuming valuable time and resources. Physical document storage became a constant challenge such as overflowing filing cabinets and difficulty in tracking documents, putting staff managing these documents at risk of occupational ergonomic hazards (Figure 3). The volume of papers not only occupy office space but also create unnecessary anxiety over lost or damaged documents, making the process of retrieving records in the future more challenging.

It was estimated that a full set of documents sent by each practitioner upon completion of the gazettement process would weigh between 0.3 to 2.5 kg per submission. With an average of 900 to 1,000 submissions per year, the estimated weight of documents would reach about 600 kg per year. This would mean that in just five years, the total weight of documents would reach about 3,000 kg, or equivalent to the average weight of an adult African elephant. This is to illustrate the physical challenges faced by the staff in handling these documents. Potential audits and litigation of SWE in the future has made the process more demanding especially in record keeping and proper documentation. Communication and coordination between units also depended on manual handovers. Whenever a hospital officer, clinical supervisor, or headquarters team raised a query, they had to return documents by postal mail or courier, often delaying the process by days or even weeks. This lack of real-time visibility on documents tracking forced staff at each stage to keep track status updates by phone or e-mail, making the process more time-consuming.

The manual verification mechanism especially on logs of clinical procedures and encounters through the logbooks also posed errors and compliance risks. Manual checking of documents led to risk of oversight. Inconsistent final assessment criteria and inconclusive supervisor narrative report opened the risk to potential disputes and re-submission or re-assessment. The overall potential risks in managing SWE are significant and must be addressed through targeted improvement measures to protect and uphold the integrity of the process.

**Figure 4:**
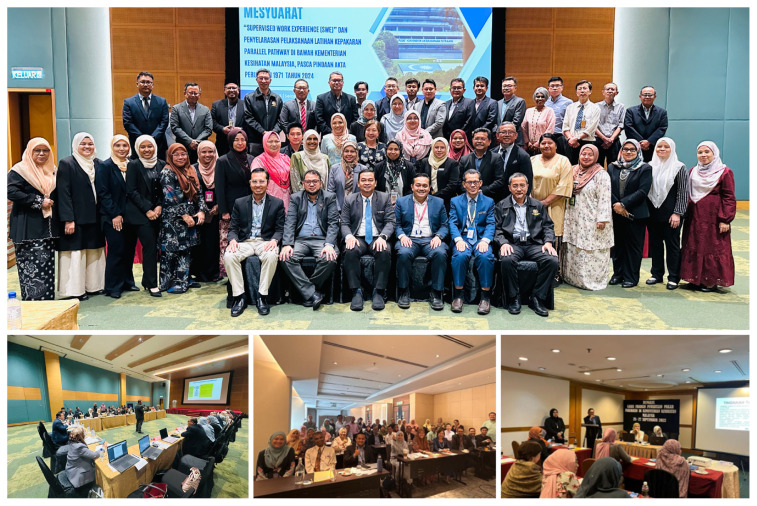
Consultative sessions on streamlining the process of SWE, specialist gazettement and e-logbook (anti clockwise from above); picture 1, group photo with heads of specialty during an engagement on SWE following the amendment of the Medical Act 1971; picture 2, discussion with heads of services on streamlining the process of SWE; picture 3, group photo with heads of specialty and technical working group on e-logbook; picture 4, discussion on development of guideline on specialist gazettement process prior to amendment of the Medical Act 1971

In response to the potential risks as described above, the Medical Development Division took the initiative to develop an online platform or e-Logbook initially intended to digitalise the specialist gazettement process which has now been extended its used to SWE. The e-Logbook was developed through a structured, consultative process between August and December 2023 (Figure 4). It began with identifying core training requirements across specialties, followed by collaborative workshops with heads of specialty and their teams to define key competencies, assessment formats, and logbook entries. This has culminated in the digitisation of logbook templates within the MPIS platform.

During the SWE, the e-Logbook serves as a record of every mandatory procedure and clinical encounter, automatically updating a real-time dashboard that both trainees and supervisors can consult at any moment. Each entry must be co-signed by a clinical supervisor, embedding performance appraisal into daily practice and capturing feedback on technical skills. At the midpoint of SWE, the system mandates a formal mid-term review, prompting supervisors to highlight learning gaps and initiate targeted intervention. This continuous feedback cultivates a more transparent and equitable assessment process, ensuring each trainee benefits from clear guidance and documented progress throughout the SWE.

**Figure 5:**
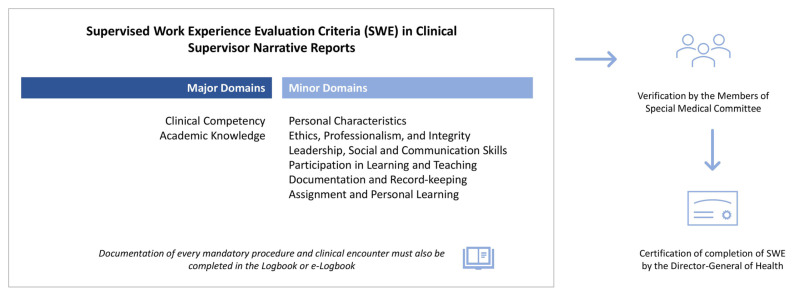
Summary of components in the supervisor narrative report for certification of completion of SWE in the Ministry of Health

Upon fulfilling all procedural requirements, supervisors consolidate their observations into a narrative report structured around eight critical domains (Figure 5). Clinical competency and academic knowledge bear the greatest weight, underscoring the important components of hands-on proficiency and theoretical strength. The remaining six domains are i) personal characteristics; ii) ethics, professionalism, and integrity; iii) leadership, social and communication skills; iv) participation in learning and teaching; v) documentation and record-keeping; and vi) assignment and personal learning. This balanced framework promotes a comprehensive appraisal, measuring not only technical capabilities but also interpersonal interactions, ethical conduct, professionalism and the capacity for lifelong learning.

The exit criteria for the programme begin with the complete documentation of every mandatory clinical procedure and clinical encounter in the e-Logbook. Each entry must be verified and signed off by the trainee’s clinical supervisor, ensuring real-time tracking and accountability. Only when all procedural requirements are logged and endorsed can the trainee proceed to the final assessment phase. To meet the exit standard, trainees must attain an overall narrative score of at least 70% and secure a minimum of 50% in each individual domain. Such threshold requirement cultivates a more comprehensive assessment as each practitioner must develop strength in all domains.

Final sign-off by the primary clinical supervisor, based on these criteria, would then allow the hospital director’s office to furnish other administrative requirements before the e-Logbook is then sent to the Ministry of Health headquarters for further verification and certification process by the members of the Special Medical Committee or JKP and subsequently by the Director General of Health.

A phased pilot roll-out of the e-Logbook ensured user readiness and smooth change management. From January to September 2024, the e-Logbook trials were conducted at Hospital Tengku Ampuan Rahimah Klang, a state hospital in Selangor that revealed key usability insights and allowed system enhancement through agile development approach. Training-of-Trainer’s sessions were then cascaded across zones such as Central Zone in October 2024, Southern and East Coast in early 2025, Northern in July 2025, and Sabah and Sarawak by the end of 2025, targeting January 2026 for nationwide launch. Through these structured trainings, hospital human resource officers and clinical supervisors could be empowered to become champions, and all necessary interventions could be done through continuous engagement.

By shifting every clinical entry, mandatory procedure, leave record, and supporting document into the e-Logbook, the system is expected to eliminate a huge volume of paper handling. The cumulative five years handling of 3,000 kg of papers may save up to 51 trees. Centralised, real-time dashboards would replace postal couriers and manual spreadsheets, giving hospitals, supervisors, and headquarters instant visibility into each trainee’s progress. Automated supervisor verification and midterm reviews promote close monitoring mechanism into SWE workflows, reducing oversight, errors and compliance risks. Standardised narrative reports with weighted scoring system across eight domains ensure consistency in final assessments, while audit trails and document tracking safeguard against lost or disputed records. Such digitalisation initiatives could transform an administrative burden into a more transparent, efficient, and accountable process workflow.

## Conclusion

The transformation of Malaysia’s specialist recognition process particularly within the public sector through legal, financial, and digital reforms is consistent with core principles of good governance particularly in the public sector. The legal shift from administrative specialist gazettement process to statutory requirement of SWE reinforce the importance of rule of law, ethical accountability, integrity of processes, and regulatory compliance, in line with core principles of good governance (11) outlined by international frameworks such as “Good Governance in The Public Sector” developed jointly by the Chartered Institute of Public Finance and Accountancy (CIPFA) and the International Federation of Accountants (IFAC) in 2014. Such transformation aligns with the Malaysian Government’s call for all public sector agencies to strengthen public sector governance through a directive issued by the Prime Minister Department, “Arahan YAB Perdana Menteri No. 1 Tahun 2023: Gerakan Pemantapan Tatakelola Nasional.”

The directive calls for public sector agencies to identify, analyse, and resolve governance issues, which include outdated or conflicting policies and laws (12). It also encourages proactive identification of systemic weaknesses, including obsolete regulations and unclear mandates, which often necessitate legal and policy reform to address root causes. The legal and financial transformation in the process of SWE would address some of those concerns and recommendations by the Government and resolve key issues such as lack of clarity in regulatory provisions. Digital deployment of e-Logbook would transform a previously paper-heavy process into a more streamlined and auditable system. This transformation not only reduces administrative burden and compliance risks but also strengthens process and data integrity, and service delivery that aligns with IFAC-CIPFA’s risk management principles. These reforms may position the Ministry of Health’s approach as a framework of integrated governance and operational efficiency to safeguard the quality of service, patients’ safety and public trust.
